# Rectal Microbiota Associated With *Chlamydia trachomatis* and *Neisseria gonorrhoeae* Infections in Men Having Sex With Other Men

**DOI:** 10.3389/fcimb.2019.00358

**Published:** 2019-10-18

**Authors:** Camilla Ceccarani, Antonella Marangoni, Marco Severgnini, Tania Camboni, Luca Laghi, Valeria Gaspari, Antonietta D'Antuono, Claudio Foschi, Maria Carla Re, Clarissa Consolandi

**Affiliations:** ^1^National Research Council, Institute of Biomedical Technologies, Milan, Italy; ^2^Department of Health Sciences, University of Milan, Milan, Italy; ^3^Microbiology, DIMES, University of Bologna, Bologna, Italy; ^4^Department of Agro-Food Science and Technology, Centre of Foodomics, University of Bologna, Cesena, Italy; ^5^Dermatology, St. Orsola-Malpighi Hospital, Bologna, Italy

**Keywords:** rectal microbiome, MSM, *Chlamydia trachomatis*, *Neisseria gonorrhoeae*, microbiota, HIV

## Abstract

*Chlamydia trachomatis* (CT) and *Neisseria gonorrhoeae* (NG) represent the most common agents of sexually transmitted rectal infections among men having sex with other men (MSM). In this study, we assessed the bacterial composition of the rectal microbiota associated with CT and/or NG infections in a cohort of men reporting unsafe rectal intercourse. A total of 125 rectal swabs were collected and four groups were compared: non-infected subjects (*n* = 53), patients with CT (*n* = 37), or NG rectal infection (*n* = 17) and patients with contemporary positivity for CT/NG (*n* = 18). CT and NG infections were detected by a real-time commercial test and the rectal microbiota composition was analyzed from rectal swabs through sequencing of the hypervariable V3-V4 regions of the 16S rRNA gene. The rectal microbiota of all subgroups was dominated by *Prevotellaceae, Enterobacteriaceae*, and *Ruminococcaceae* families. Irrespective of the analyzed subgroup, we found that the rectal environment of all the enrolled MSM was rich in *Prevotella* and *Escherichia* genera. Moreover, a shift in the bacterial composition between patients with sexually transmitted rectal infections and controls was noticed: infected patients were characterized by a depletion of *Escherichia* species, associated with an increase of anaerobic genera, including *Peptoniphilus, Peptostreptococcus*, and *Parvimonas*. Overall, the presence of rectal symptoms did not significantly modify the rectal microbiota profiles among the four groups of analyzed patients. We confirmed that HIV-positive patients are characterized by a lower bacterial richness than HIV-negative subjects. However, we found that the presence of HIV has a different impact on bacterial rectal communities compared to CT and NG infections, modifying the relative abundance of several genera, including *Gardnerella, Lactobacillus, Corynebacterium*, and *Sutterella*. Information about the rectal microbiota composition in CT and NG infections could shed light on the pathogenesis of these conditions and could contribute to the onset of new strategies for their control.

## Introduction

*Chlamydia trachomatis* (CT) and *Neisseria gonorrhoeae* (NG) are the etiological agents of the most common sexually transmitted rectal infections (STIs) in men having sex with other men (MSM) (Danby et al., 2016; Tao et al., 2016).

These infections are often asymptomatic, acting as a significant reservoir for their further spread; if left untreated, they can lead to several sequelae and complications (Grov et al., 2016; Foschi et al., 2018a). Moreover, missed rectal infections could contribute to the onset of alarming multi-drug resistance in *N. gonorrhoeae* (Unemo and Shafer, 2014). In addition, chlamydial and gonococcal rectal infections are associated with an increased risk of HIV infection transmission (Barbee et al., 2017).

To the best of our knowledge, no information about the composition of the rectal microbiota during ongoing *C. trachomatis* or *N. gonorrhoeae* infections is currently available. The characterization of the bacterial environment of the rectal mucosa, where chlamydial and gonococcal infections originate and proliferate, could be crucial to better understand the pathogenesis of these rectal STIs.

Several studies comparing fecal collection and rectal biopsies for microbiome analysis show that the intestinal lumen and mucosa can be colonized by distinct microbial communities (Araújo-Pérez et al., 2012; Tang et al., 2015). The mucosal microbiota is in close contact with the intestinal epithelium, thus interacting more directly with the host immune system than the fecal bacteria (Tang et al., 2015). As a consequence, mucosal microbiota is possibly linked to disease development (Goto and Kiyono, 2012). Moreover, there are significant differences in terms of nutrient availability between the epithelial mucus layer and the gut lumen environment (Igartua et al., 2017). Fecal samples reflect what is held in the luminal environment, including bacteria ingested with food, whereas direct sampling of the intestinal mucosa is more representative of the interactions between the endogenous microbiome and the host (Ingala et al., 2018).

Biopsies are able to capture the diversity of microbial populations in the mucosal layer, where adherent bacteria reside (Sonnenburg et al., 2004). Unfortunately, procedures for obtaining colorectal biopsies (i.e., sigmoidoscopy, anoscopy, or colonoscopy) are expensive, time-consuming and invasive, often leading to patient discomfort. Previous works have shown that rectal swabs are suitable to assess the characteristics of the gut microbiota (distal gastrointestinal tract), being an appropriate alternative to mucosal biopsy specimens (Budding et al., 2014; Bansal et al., 2018; Zhang et al., 2018). In this context, it has been hypothesized that microbiota profiles derived from rectal swabs can be used for clinical diagnostics and large-scale studies (Budding et al., 2014).

The bacterial composition of the ano-rectal mucosa has been previously studied in different conditions. As an example, Kelley et al. demonstrated that MSM engaging in condomless receptive anal intercourse harbor a peculiar rectal mucosal environment, characterized by an enrichment of *Prevotellaceae* and a depletion of *Bacteroidaceae* (Kelley et al., 2017).

In this study, we assessed the bacterial community profiles of the rectal microbiota associated to CT and/or NG infections by analyzing rectal swabs collected from a cohort of MSM. These data could be useful to set up new diagnostic/prognostic tools, to find correlations with the presence of peculiar clinical or behavioral aspects (e.g., rectal signs/symptoms, onset of complications, etc.), as well as to evaluate the possibility of a different susceptibility to STIs based on the rectal microbiota composition. It follows that intriguing perspectives for the control of rectal CT and NG infections, in terms of prevention and treatment, could be opened.

## Materials and Methods

### Study Population and Sample Collection

Eligible patients were selected from a group of MSM attending the STI Outpatients Clinic of S. Orsola-Malpighi Hospital in Bologna (Italy) and reporting unsafe rectal intercourse. Exclusion criteria were: being under the age of 18 years; antibiotic treatments in the month before the study; presence of inflammatory bowel diseases (IBD); presence of infectious intestinal pathologies; use of enemas within 3 days before sampling. Moreover, samples positive for *Mycoplasma genitalium*, HSV and *Treponema pallidum* rectal infections were further excluded from the study.

In particular, *M. genitalium* rectal infections were not included in the study as a group apart, despite their growing and well-recognized importance (Bissessor et al., 2016; Foschi et al., 2018a), because of the low number of cases found during the study period (i.e., 5 single *M. genitalium* infections and 2 *N. gonorrhoeae*/*M. genitalium* co-infections).

For all the patients reporting gastrointestinal symptoms (e.g., diarrhea, tenesmus, etc.), the presence of an infectious gastroenteritis was excluded by means of microscopic (i.e., stool microscopy for protozoa and helminths), culture-based (i.e., stool cultures for pathogenic *Salmonella, Shigella, Campylobacter*, and *Yersinia* species), serological (i.e., anti-HAV IgM antibodies), or molecular approaches (i.e., stool PCR for adenovirus, rotavirus, norovirus, *Clostridium difficile* toxins, and *Escherichia coli* pathotypes).

Personal data and information about ano-rectal symptoms were recorded for each patient. Afterwards, a clinical examination was carried out, evaluating the perianal skin for the presence of lesions (e.g., ulcers, condylomas). No anoscopy was performed.

An ano-rectal swab (E-Swab, Copan, Brescia, Italy) for the molecular detection of *C. trachomatis, N. gonorrhoeae, M. genitalium*, HSV, and *T. pallidum* was collected from each patient. The adequacy of rectal mucosal sampling in terms of cellularity degree was confirmed by means of PCR, targeting the human beta-globin gene (Mujugira et al., 2015).

Clinical and microbiological data about HIV infection were recorded, under the patient's consent. All patients were managed following the regular STIs evaluation of the Clinic: (i) serological screening for HIV in previously negative subjects or in those who had never performed it; (ii) monitoring through RNA viral loads, CD4 cells count and CD4/CD8 ratio for patients with known HIV infection.

Peripheral blood CD4 and CD8 lymphocytes were counted by flow cytometry (FACScan, Becton and Dickinson, Mountain View, CA, USA). HIV serology was performed with Architect HIV Ag/Ab Combo assay (Abbott, Wiesbaden, Germany) as a screening test, using VIDAS HIV DUO Quick assay (bioMerieux, Marcy l'Etoile, France) and INNO-LIA HIV I/II Score (Innogenetics, Gent, Belgium) as confirmatory tests. HIV-RNA viral load measurements were performed by a real-time PCR assay (COBAS AmpliPrep/COBAS TaqMan HIV-1 Quantitative Test; Roche Molecular Systems, Pleasanton, CA, USA).

On the basis of microbiological results, eligible patients were allocated in one of the following groups: “no rectal infection” (negativity for rectal CT and NG), “CT” (positivity for CT, irrespective of the serovar), “NG” (positivity for NG), and “CT/NG” (contemporary positivity for rectal CT and NG).

The study protocol was reviewed and approved by the Ethical Committee of St. Orsola-Malpighi Hospital (78/2017/U/Tess). A written informed consent to the work was collected from all subjects.

### Diagnosis of Rectal Infections and *C. trachomatis* Typing

Ano-rectal swabs were processed by Versant CT/GC DNA 1.0 Assay (Siemens Healthineers, Tarrytown, NY, USA), a duplex real-time PCR test detecting the presence of CT and/or NG DNA, as described in Marangoni et al. (2015).

Starting from the remaining DNA eluate of the Versant PCR plate, each sample was tested for *M. genitalium* with an in-house quantitative PCR assay, as previously described (Foschi et al., 2018b).

Rectal HSV and *T. pallidum* infections were excluded by means of a multiplex molecular approach (FTD genital ulcer, Fast Track Diagnostics, Esch sur Alzette, Luxembourg).

Serovar identification of CT-positive samples was achieved by an *omp1* gene semi-nested PCR followed by RFLP analysis, as described in Foschi et al. (2018c).

### Analysis of the Rectal Microbiota

Hypervariable V3-V4 regions of the bacterial 16S rRNA gene from rectal swab genomic DNA were amplified. PCR conditions as well as primer sequences were retrieved from Illumina 16S Sample Preparation Guide (https://support.illumina.com/documents/documentation/chemistry_documentation/16s/16s-metagenomic-library-prep-guide-15044223-b.pdf) (Illumina, San Diego, CA, USA); primer selection was originally described in Klindworth et al. (2013). Indexed libraries were prepared by equimolar (4 nmol/L) pooling and sequenced on Illumina MiSeq platform with a 2 × 300 bp run, according to manufacturer's instructions (Illumina).

The 16S rRNA raw sequences were merged using Pandaseq (Masella et al., 2012), then low quality reads (i.e., showing stretches of bases with a Q-score <3 for more than 25% of their length) were discarded. A subset of 50,000 reads for each sample was randomly extracted, in order to obtain a similar number of reads for each sample. Bioinformatic analyses were conducted using the QIIME pipeline (release 1.8.0; Caporaso et al., 2010), clustering filtered reads into Operational Taxonomic Units (OTUs) at 97% identity level. Taxonomic assignment was performed via the RDP classifier (Wang et al., 2007) against the Greengenes database (release 13.8; (ftp://greengenes.microbio.me/greengenes_release/gg_13_8_otus), with a 0.5 identity threshold.

Biodiversity and distribution of the microorganisms were characterized via alpha- and beta-diversity evaluations. Alpha-diversity was measured using Chao1, observed species, Shannon diversity, Good's coverage and Faith's phylogenetic diversity (PD_whole_tree) metrics; statistical evaluation among alpha-diversity indices was performed by a non-parametric Monte Carlo-based test, using 9,999 random permutations. Weighted and unweighted UniFrac distances and permanova (“adonis” function) in the R package “vegan” (version 2.0–10; Oksanen et al., 2013) were used to compare the microbial community structure in beta-diversity analysis.

### Statistical Analysis

Differences in clinical and demographic parameters were tested by Chi-square test or ANOVA test, using Prism 5.02 version for Windows (GraphPad Software, San Diego, CA, USA).

Unless otherwise stated, *p* < 0.05 were considered as significant for each statistical analysis.

For each phylogenetic level, only taxa present at >1% average relative abundance in at least one of the experimental categories, were considered, in order to focus on the major players of the rectal microbiota. Differences in abundances of bacterial taxa among experimental groups were analyzed by Kruskal-Wallis followed by Dunn's *post-hoc* tests, and applying a Benjamini-Hochberg correction for multiple testing, using MATLAB software (Natick, MA, USA). Due to the exploratory nature of our experiments, a FDR threshold <0.15 was chosen in order to not miss any potentially relevant difference in bacterial groups. Since the correction for multiple comparisons did not change the significance of taxonomic data results, for clarity, uncorrected *p*-values were reported in the text.

Correlation between bacterial genera and CD4/CD8 ratio or viral loads for HIV-positive individuals was calculated via the Pearson's correlation coefficient; only correlations showing a *p*-value of the linear model <0.05 were reported.

To evaluate the respective contributions of HIV and CT or NG infections on rectal microbial changes, a two-way ANOVA was conducted in R, by taking advantage of the “aov” function of the R package “stats” (Chambers et al., 2017), applied to take into consideration type II errors. For this purpose, a cut-off *p*-value of 0.05 was accepted. Variables that were not-normally distributed were transformed according to Box and Cox (2018).

## Data Availability

Raw reads are available in NCBI Short Read Archive (SRA, http://www.ncbi.nlm.nih.gov/sra) under accession number PRJNA545872.

## Results

### Study Population

A total of 151 MSM were, at first, included in the study, but 26 of them were, subsequently, excluded due to low quantity of raw sequencing reads (i.e., <20,000). The final dataset consisted of 125 samples, divided into 4 groups: negative subjects (“no infection,” *n* = 53), patients with CT rectal infection (“CT,” *n* = 37), patients with NG rectal infection (“NG,” *n* = 17), and patients with concurrent positivity for CT and NG (“CT/NG,” *n* = 18). The 26 excluded samples were composed by 8 non-infected, 3 CT, 14 NG, and 1 CT/NG patients. For the final dataset, the average number of obtained sequences was of 44,091 ± 9,764 reads (range: 20,407–50,000 reads).

To evaluate whether the number of subjects included was sufficient to detect significant variations between the groups, a sample size calculation was performed, using existing data regarding the shift of the rectal mucosal microbiota during HIV infection (Nowak et al., 2017). In particular, we focused on several bacterial genera that differed significantly between HIV-negative and HIV-positive treated subjects. With a significance level of 0.05 and a power of 80%, we determined that 17 patients per group would be sufficient to detect significant variations in the rectal microbiota composition between the different experimental conditions.

Details regarding primary demographic and clinical characteristics of the enrolled subjects are shown in Table 1. All the patients denied receptive anal intercourse, as well as the use of lubricants in the 3 days prior to the enrollment. During ano-genital examination, no ulcers, warts, or herpetic lesions were noticed.

**Table 1:** Primary demographic and clinical characteristics of the enrolled subjects.

	**No infection (*n* = 53)**	**CT (*n* = 37)**	**NG (*n* = 17)**	**CT/NG (*n* = 18)**	***p*-value**
**Mean age (years** **±*****SD*****)**	34.4 ± 9.1	34.0 ± 9.0	31.0 ± 9.3	30.8 ± 10.4	0.48
**Rectal symptoms**	0/53 (0.0%)	24/37 (64.8%)	3/17 (17.6%)	7/18 (38.9%)	<0.0001
Rectal bleeding	–	2/24 (8.3%)	0/3 (0.0%)	1/7 (14.2%)	
Rectal discharge	–	14/24 (58.3%)	2/3 (66.6%)	3/7 (42.8%)	
Tenesmus	–	4/24 (16.6%)	0/3 (0.0%)	2/7 (28.5%)	
Diarrhea	–	2/24 (8.3%)	1/3 (33.3%)	0/7 (0.0%)	
Ano-rectal pain	–	6/24 (25.0%)	1/3 (33.3%)	4/7 (57.1%)	
**HIV infection**					
Yes	11/53 (20.7%)	21/37 (56.7%)	3/17 (17.6%)	5/18 (27.7%)	0.0002
No	40/53 (75.5%)	14/37 (37.8%)	11/17 (64.8%)	12/18 (66.6%)	
Unknown	2/53 (3.8%)	2/37 (5.4%)	3/17 (17.6%)	1/18 (5.5%)	
CD4/CD8 ratio	0.77 ± 0.3	0.79 ± 0.5	0.94 ± 0.4	0.82 ± 0.4	0.94
(Mean ±*SD*; min–max)	(0.35–1.48)	(0.27–2.62)	(0.51–1.36)	(0.34–1.37)	
Viral load (copies/mL)	36,176 ±	203,714 ±	15,777 ±	289 ±	0.48
(Mean ±*SD*)	109,609	480,317	27,318	642	
**CT serovars**					
LGV (L2 serovar)	–	20/37 (54.0%)	–	6/18 (33.3%)	-
Non-L serovars					
D	–	7/37 (18.9%)	–	3/18 (16.6%)	
E	–	5/37 (13.5%)	–	4/18 (22.2%)	
F	–	0/37 (0.0%)	–	1/18 (5.5%)	
G	–	4/37 (10.8%)	–	2/18 (11.1%)	
H	–	1/37 (2.7%)	–	2/18 (11.1%)	

It is worth to underline that the presence of rectal symptoms was mainly found in patients with a single chlamydial infection (64.8%). This aspect was associated with the high proportion of cases of lymphogranuloma venereum infection (LGV; L2 serovar by CT molecular typing) found in this group (54.0%). Indeed, 80.0% (16/20) of LGV cases were characterized by various ano-rectal symptoms (e.g., anal pain, rectal discharge, bleeding), in contrast to the 47.1% (8/17) of patients with non-L serovar infections. Non-L CT cases (29) were mainly due to serovars D (34.5%) and E (31.0%), followed by serovars G (20.7%), H (10.3%), and F (3.5%). In the whole dataset, a total of 40 subjects (40/125; 32.0%) were HIV-positive. These cases were mainly found in the group of patients with CT rectal infections, with a significant association between the presence of HIV and chlamydial L2 serovars (*p* = 0.0003). Most of the HIV-positive patients (27; 67.5%) were characterized by a well-controlled infection, with undetectable or very low viral loads (<20 RNA copies/mL). The CD4/CD8 ratio ranged between 0.27 and 2.62, with a mean of 0.80 ± 0.46.

### Rectal Microbiota Structure Characterization

**Figure 1:**
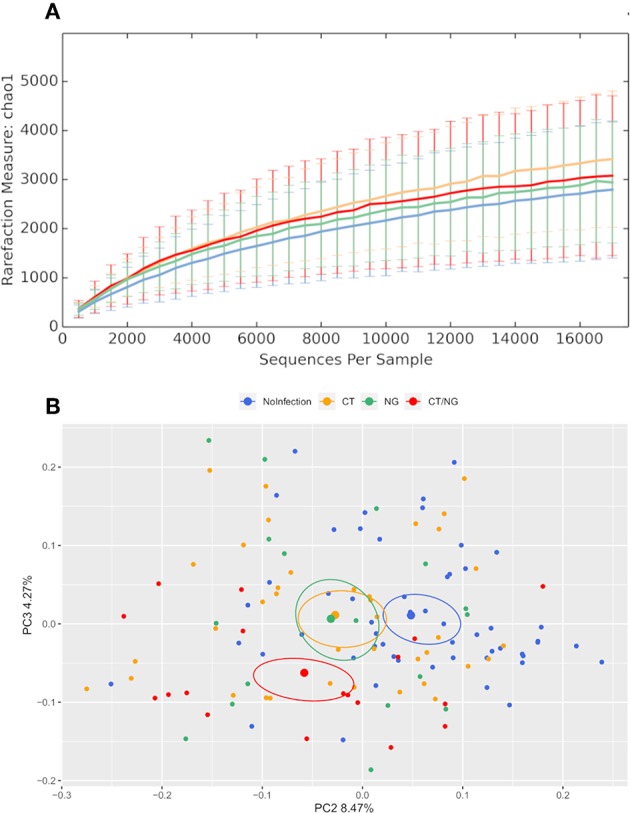
Structure of the rectal microbiota. Microbial composition from rectal swabs of the different analyzed groups: control subjects (no infection, blue), patients with *C. trachomatis* (CT, yellow) infection, patients with *N. gonorrhoeae* infection (NG, green), and subjects positive contemporary for both pathogens (CT/NG, red). **(A)** Alpha-diversity rarefaction curves of Chao1 index. **(B)** Principal Coordinates Analysis (PCoA) plot based on unweighted Unifrac distance (beta-diversity). Each point corresponds to a sample. For each experimental group, the SEM-based confidence ellipse and the average value centroid are depicted. The second and third principal coordinates are represented.

Alpha-diversity analysis showed significant differences in biodiversity of infected subjects grouped together (Observed Species, *p* = 0.035; Shannon, *p* = 0.009). In particular, all three groups with rectal infections were characterized by a slightly higher bacterial diversity compared to controls (Figure 1). Comparisons between each experimental groups and uninfected patients did not show any statistical significance; detailed data for all the metrics (Chao1, observed species, Shannon diversity, Good's coverage, and Faith's phylogenetic diversity) are reported in Table S1.

Principal coordinates analysis (PCoA) showed that infected subjects were significantly separated from patients without rectal infections, both grouping all infected patients together (*p* < 0.01 for both weighted and unweighted distances) and comparing each group with controls (for all pairwise comparisons: unweighted Unifrac, *p* < 0.03; non-infected subjects vs. CT, weighted Unifrac, *p* = 0.01) (Figure 1 and Figure S1), highlighting significant differences in the composition of rectal microbiota. Within the group with single CT rectal infection, we found significant differences in beta-diversity between L2 (*n* = 20) and non-L (*n* = 17) CT serovars (*p* = 0.01 for both weighted and unweighted distances).

Overall, the presence of rectal symptoms, as well as the age of patients, did not significantly modify the rectal microbiota profiles among the four groups of patients analyzed. Indeed, no significant differences were found when comparing symptomatic *vs*. asymptomatic subjects within the different groups (data not shown). Comparing uninfected controls (*n* = 53) with all the asymptomatic infected patients (*n* = 38), significant differences in alpha and beta-diversity were found, indicating that the presence of CT or NG infection, not of the symptoms, was responsible for microbial changes.

**Figure 2:**
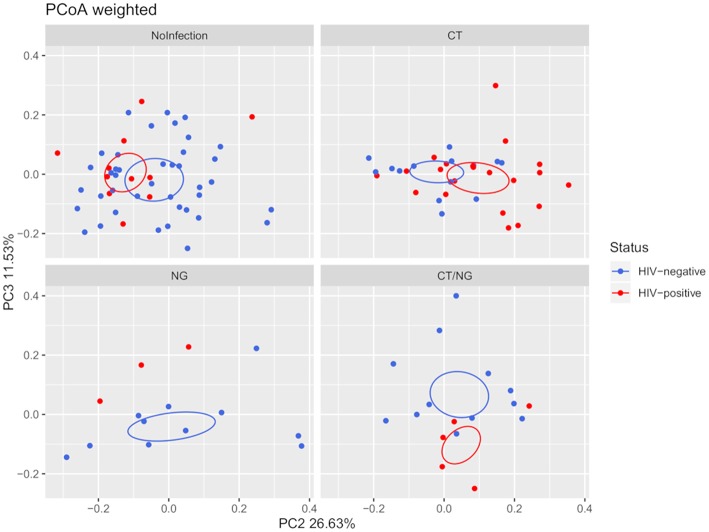
Principal Coordinates Analysis (PCoA) plot regarding HIV infection per group, based on weighted Unifrac distance (beta-diversity). For the HIV-status of each experimental group, a SEM-based confidence ellipse is depicted. The second and third principal coordinates are represented.

Moreover, HIV-negative subjects showed a higher bacterial diversity compared to HIV-positive ones (Shannon's index, *p* = 0.039) within non-infected subjects. Analyzing together the three groups of infected subjects, a significant difference in beta-diversity (weighted Unifrac, *p* = 0.05; unweighted, *p* = 0.02) was found in the comparison between HIV-positive (*n* = 29) and HIV-negative (*n* = 37) patients. However, as shown in Figure 2, within the four groups of subjects, the significance was retained only for patients positive for single *C. trachomatis* rectal infection (HIV-positive vs. HIV-negative: unweighted, *p* = 0.02; weighted, *p* = 0.03).

### Taxonomic Composition of Rectal Bacterial Communities

**Figure 3:**
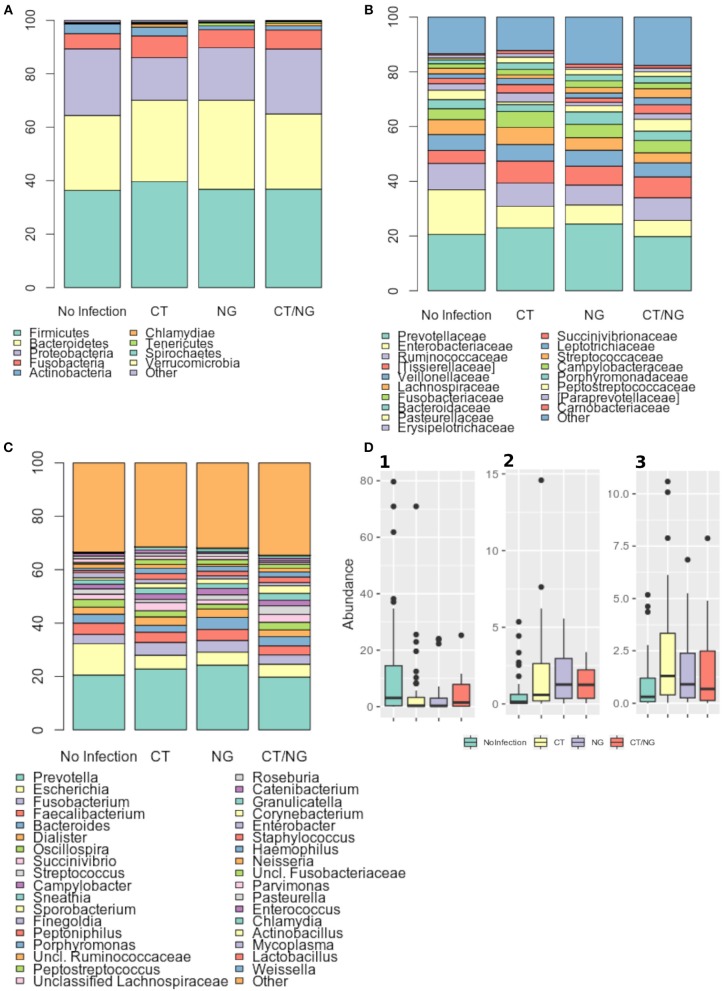
Taxonomic composition of the rectal microbiota. Stacked bar charts of taxonomy relative abundances at **(A)** phylum, **(B)** family, and **(C)** genus level for the different subgroups. Only phyla, families and genera present at relative abundances >1% on average in at least one subgroup are reported. Remaining taxa are grouped in the “Other” category. **(D)** Boxplots of the relative abundances of (1) *Escherichia* (2) *Peptostreptococcus* (3) *Peptoniphilus* genera in the different subgroups considered.

At phylum level, all samples were characterized by Firmicutes, Bacteroidetes, and Proteobacteria (Figure 3A), with no significant differences in their abundances across groups. Firmicutes and Bacteroidetes dominated the rectal microbiota of all MSM groups, with relative abundance (rel. ab.) ranging from about 36–39% and from about 28–33%, respectively. Fusobacteria and Actinobacteria were less represented in the rectal microbiota composition, never exceeding ~10% of rel. ab. in all the groups (Table S2).

As for the family level, *Prevotellaceae* (rel. ab., 19–24%), *Enterobacteriaceae* (7–16%), and *Ruminococcaceae* (7–9%) were the most abundant groups. *Enterobacteriaceae, Tissierellaceae, Peptostreptococcaceae, Carnobacteriaceae*, and *Leuconostocaceae* were found significantly different (*p* ≤ 0.02, Kruskal-Wallis test for all taxa) (Figure 3B and Table S3). In particular, patients with single CT and NG infections were both characterized by significant lower levels of *Enterobacteriaceae* (*p* = 0.006 and *p* = 0.01, respectively) and higher amounts of *Peptostreptococcaceae* (*p* = 0.0001 and *p* = 0.0002, respectively) compared to non-infected controls. On the other hand, in subjects with a contemporary CT/NG rectal infection, we observed a decreasing trend for *Enterobacteriaceae* (*p* = 0.09), while *Tissierellaceae, Peptostreptococcaceae, Carnobacteriaceae*, and *Leuconostocaceae* were found significantly more relatively abundant compared to controls (*p* ≤ 0.01).

At genus level, *Prevotella* was the most represented genus in all groups, followed by *Escherichia* (Figure 3C). Patients with single CT and NG infections exhibited lower levels of *Escherichia* species compared to non-infected individuals (*p* = 0.002 and *p* = 0.01, respectively) (Table 2 and Figure 3D). In all infected patients, this observation was associated with a significantly increased relative abundance of several anaerobic genera, such as *Peptoniphilus, Peptostreptococcus*, and *Parvimonas* (*p* < 0.05) (Figure 3D). Subjects with a contemporary CT/NG positivity were also characterized by significantly higher levels of *Granulicatella*, unclassified *Fusobacteriaceae*, and *Pasteurella* (*p* ≤ 0.012 in all tests), the latter being increased also in NG single-infection group (*p* = 0.005).

**Table 2:** Average relative abundance of main microbial genera.

	**Average (stdev)**				**Kruskal-Wallis/Dunn's test**			
	**No infection**	**CT**	**NG**	**CT/NG**	**K-W *p*-value**	**CT**	**NG**	**CT/NG**
*Prevotella*	20.54 (11.63)	22.81 (10.31)	24.24 (7.92)	19.76 (9.78)	–	–	–	–
*Escherichia*	11.72 (18.33)	5.15 (12.77)	4.88 (8.99)	4.86 (6.61)	0.016	0.002	0.01	–
*Fusobacterium*	3.50 (5.64)	4.79 (5.31)	4.39 (5.65)	3.50 (3.70)	–	–	–	–
*Faecalibacterium*	4.22 (4.65)	3.86 (3.54)	4.13 (5.36)	3.38 (3.33)	–	–	–	–
*Bacteroides*	3.33 (4.32)	2.54 (3.82)	4.50 (4.92)	3.45 (5.67)	–	–	–	–
*Dialister*	2.65 (2.60)	3.18 (2.13)	3.13 (1.75)	2.51 (1.93)	–	–	–	–
*Oscillospira*	2.94 (3.63)	2.28 (2.05)	1.82 (2.07)	2.85 (3.82)	–	–	–	–
*Streptococcus*	2.02 (4.90)	1.23 (2.36)	1.92 (3.51)	3.30 (6.37)	–	–	–	–
*Campylobacter*	1.67 (2.81)	2.06 (2.60)	2.42 (2.49)	2.07 (2.73)	–	–	–	–
*Sneathia*	1.60 (4.72)	2.20 (4.28)	1.77 (2.79)	2.55 (5.82)	–	–	–	–
*Sporobacterium*	0.88 (1.86)	1.78 (3.51)	1.79 (3.26)	2.88 (4.46)	–	–	–	–
*Finegoldia*	1.83 (2.55)	1.48 (1.79)	1.19 (1.85)	1.16 (1.44)	–	–	–	–
*Peptoniphilus*	0.85 (1.21)	2.12 (2.14)	1.70 (2.16)	2.08 (3.14)	0.002	<0.001	–	0.01
*Porphyromonas*	0.87 (1.69)	2.01 (3.63)	1.79 (2.75)	1.90 (2.70)	–	–	–	–
*Peptostreptococcus*	0.60 (1.11)	1.92 (2.86)	1.83 (1.83)	1.42 (1.13)	<0.001	<0.001	0.001	<0.001
*Roseburia*	0.96 (1.30)	1.14 (1.55)	1.17 (2.15)	0.63 (0.95)	–	–	–	–
*Granulicatella*	0.48 (0.94)	1.11 (2.14)	1.23 (2.52)	1.00 (1.74)	0.022	0.009	–	0.005
Corynebacterium	1.73 (4.41)	0.97 (2.01)	0.26 (0.39)	0.35 (0.57)	–	–	–	–
Enterobacter	1.35 (4.76)	1.09 (5.44)	0.31 (1.21)	0.40 (1.63)	–	–	–	–
*Staphylococcus*	2.35 (6.36)	0.72 (2.48)	1.38 (4.86)	0.66 (1.70)	–	–	–	–
*Haemophilus*	2.12 (5.09)	0.64 (1.34)	0.71 (1.58)	3.91 (10.20)	–	–	–	–
*Neisseria*	0.06 (0.35)	0.00 (0.01)	4.82 (7.11)	7.09 (14.75)	<0.001	–	<0.001	<0.001
*Uncl. Fusobacteriaceae*	0.51 (1.36)	1.00 (1.53)	0.50 (0.56)	0.95 (1.98)	0.035	0.005	–	0.019
*Parvimonas*	0.40 (1.10)	0.99 (1.46)	1.15 (2.54)	0.66 (1.19)	0.002	0.001	0.003	0.01
*Pasteurella*	1.16 (4.22)	0.03 (0.08)	0.27 (0.74)	0.01 (0.03)	0.022	–	0.012	0.005
*Enterococcus*	0.63 (1.97)	0.06 (0.12)	1.12 (2.91)	0.30 (0.68)	–	–	–	–
*Chlamydia*	0.01 (0.03)	1.05 (1.85)	0.00 (0.00)	0.90 (1.87)	<0.001	<0.001	0.014	<0.001
Other	29.54	31.78	25.36	30.69				

*Data are expressed as mean ± standard deviation (stdev). For each genus, significant p-values of the non-parametric Kruskal-Wallis test and of pairwise comparison (Dunn's test) vs. non-infected patients are reported. Only the most significant genera present at relative abundances >1% on average in at least one subgroup, are listed*.

Considering the rectal microbiota profiles at genus level of HIV-positive vs. HIV-negative patients, a negative correlation between *Escherichia* and *Prevotella* genera was noticed within the control group (i.e., no infection): *Escherichia* genus tended to increase in parallel with a depletion of *Prevotella* (Figure S2).

By means of a multivariate model (two-way ANOVA), we investigated the respective contribution of HIV and CT or NG infections on the rectal microbiome changes. We found that infections had a different impact on bacterial rectal communities compared to HIV-status. Indeed, the presence of HIV affected the levels of different bacterial communities compared to rectal infections, with the exception of *Granulicatella* (Table S4).

Globally, as detailed in Table 3, HIV-positive patients were characterized by significantly higher levels of *Gardnerella, Lactobacillus*, and *Granulicatella* genera, with a depletion of *Corynebacterium, Sutterella*, and *Ruminococcus* compared to HIV-negative patients.

**Table 3:** Average relative abundance of rectal microbial communities showing significant differences between HIV-positive and negative subjects.

	**HIV-positive**	**HIV-negative**	***p*-values**
**Phylum**			
Fusobacteria	8.81 ± 9.09	5.96 ± 8.27	0.03
**Family**			
*Corynebacteriaceae*	1.00 ± 3.14	1.19 ± 3.30	0.014
*Carnobacteriaceae*	1.16 ± 0.21	0.64 ± 0.21	0.014
*Alcaligenaceae*	0.46 ± 0.57	0.89 ± 1.41	0.016
*Fusobacteriaceae*	6.10 ± 6.37	4.07 ± 5.55	0.04
**Genus**			
Unclassified *Bacteroidales*	0.36 ± 0.53	0.21 ± 0.40	0.002
*Gardnerella*	0.64 ± 1.88	0.12 ± 0.37	0.009
*Corynebacterium*	1.00 ± 3.14	1.19 ± 3.30	0.014
*Sutterella*	0.46 ± 0.57	0.88 ± 1.41	0.018
*Lactobacillus*	0.10 ± 0.42	0.27 ± 2.31	0.018
*Granulicatella*	1.15 ± 2.10	0.64 ± 1.47	0.02
*Ruminococcus*	0.37 ± 0.60	0.74 ± 0.98	0.02

*Data are expressed as mean ± standard deviation (stdev). For each phylum, family, or genus, significant p-values of the non-parametric Mann-Whitney t-test are reported*.

In HIV-infected patients, CD4/CD8 ratio was positively correlated to *Ruminococcus* (*R* = 0.313), *Sutterella* (*R* = 0.588), and *Mitsuokella* (*R* = 0.314) genera. Moreover, we found a positive correlation between HIV viral loads and *Acidaminococcus* (*R* = 0.431)*, Dorea* (*R* = 0.328)*, Actinomyces* (*R* = 0.324), and *Lachnospira* (*R* = 0.313) genera. No negative correlations were found.

## Discussion

To the best of our knowledge, this is the first report assessing the bacterial composition of the rectal environment in case of sexually transmitted infections due to *C. trachomatis* and *N. gonorrhoeae* in a group of MSM.

For this purpose, ano-rectal swabs collected from patients reporting unsafe intercourse underwent sequencing of V3-V4 regions of 16S rRNA bacterial gene. Ano-rectal specimens are non-invasive, easy-to-collect, and particularly suitable to assess the microbial characteristics of the distal gastrointestinal tract (Zhang et al., 2018).

However, with no anoscopy, this kind of sampling collects material from the entire intestinal lumen, instead of from specific sites of rectal infection (e.g., ulcers, local inflammation). Therefore, additional studies with a more targeted sampling will be needed for an in-depth understanding of the dynamics occurring during rectal infections.

First, we observed that *Prevotella* species dominated the rectal microbiota of all the subjects, irrespective of the analyzed group. These data confirmed the results of Armstrong et al. showing that the gut microbiome of MSM is more likely to be *Prevotella*-rich than that of men having sex with women (MSW) and females (3.9 times higher relative abundance of *Prevotella*) (Armstrong et al., 2018). Similarly, it has been shown that the rectal microbiota of MSM engaging in condomless receptive anal intercourse, is enriched for the family *Prevotellaceae*, probably because of the mechanical microtrauma and deposition of semen resulting in transient damages and inflammatory responses able to affect the gut commensal microbiota (Kelley et al., 2017).

Second, we noticed an interesting shift in the bacterial composition between patients with sexually transmitted rectal infections and controls. Infected patients were characterized by a depletion of *Escherichia* species, associated with increased abundances of mainly anaerobic genera, including *Peptoniphilus, Peptostreptococcus*, and *Parvimonas*.

Although further investigations are needed to understand the reasons behind these microbial changes, we can hypothesize that the inflammatory responses induced by chlamydial/gonococcal infections could affect and modify the composition of the rectal microbiota. Indeed, the oxygen consumption by the pathogens themselves or by the recruited leukocytes could favor the proliferation of strict anaerobes, as the members of the *Peptostreptococcaceae* family.

During the infection, *N. gonorrhoeae* elicits a strong inflammatory response with the release of proinflammatory cytokines and a significant influx of neutrophils (Quillin and Seifert, 2018). Despite the differences in life cycles and pathogenesis, in a similar way, *C. trachomatis* recruits natural killer (NK) cells and neutrophils to the site of infection with the production of cytokines and metalloproteinases (Vasilevsky et al., 2014). This “proinflammatory milieu” could alter the equilibrium of the bacterial communities of the rectal mucosa, with a depletion of some species.

Moreover, for their nutritional requirements, chlamydia and gonococcus could modify specific metabolic pathways, leading to the preferential proliferation of anaerobes in the rectal microbiota.

Nevertheless, we cannot rule out that the depletion of *E. coli* precedes CT or NG infection, acting as a risk factor that promotes pathogens establishment and replication on the rectal mucosa. If so, new antibiotic-free preventive approaches based on *E. coli* probiotic administration could probably be considered in the near future. It should be remembered that probiotic formulations of *E. coli* (e.g., *Escherichia coli* Nissle 1917) have been successfully employed for fighting infectious and inflammatory bowel diseases (Losurdo et al., 2015; Sonnenborn, 2016). The high inhibitory effect displayed by *E. coli* Nissle against various intestinal pathogenic bacteria and yeasts (i.e., *Salmonella*, pathogenic *E. coli, Shigella, Yersinia, Listeria, Candida*) could be potentially applied also to rectal sexually transmitted infections, such as CT and NG (Sassone-Corsi and Raffatellu, 2015).

Our data disagree with previously shown observations for other inflammatory conditions affecting the colorectal mucosa, such as *Clostridium difficile* infections and ulcerative colitis, which are characterized by higher concentrations of *Enterobacteriaceae*, primarily *E. coli*, in the rectal environment (Ohkusa and Koido, 2015; Schäffler and Breitrück, 2018). Presumably, each pathological agent or condition acts differently on the rectal microbial environment, leading to peculiar changes that can be used as specific fingerprints for diagnostic/prognostic purposes.

It is worth mentioning that the presence of rectal symptoms had no impact on the rectal microbiota profiles. This means that different factors, other than microbial environment, affect the presence of rectal symptoms (e.g., bacterial virulence, bacterial loads, age, local factors, individual susceptibility). Information about rectal inflammation grading (e.g., myeloperoxidase, proinflammatory cytokines) would help in elucidating the complex interactions between infection, immunity and microbial local communities (Heiligenberg et al., 2013).

Interesting data emerged when rectal microbiota features were looked at taking into account the HIV-status. As a first observation, we confirmed that HIV-positive patients were characterized by a lower bacterial diversity than HIV-negative subjects. Indeed, it has been previously shown that HIV infection is characterized by a reduced bacterial richness in the gut microbiome, with the depletion of some commensal species and the enrichment of a few opportunistic pathogens (McHardy et al., 2013; Noguera-Julian et al., 2016). Presumably, these microbial changes are associated with patient's immune dysfunction, considering that an early antiretroviral therapy (ART) helps to preserve gut microbial richness, normalizing the reduction in alpha-diversity (Nowak et al., 2015).

Nevertheless, other authors showed that untreated HIV infection does not significantly alter the rectal microbial composition (Nowak et al., 2017), and that several potential modifying factors should be taken into account, such as the presence and type of ART, prophylactic antibiotic treatments, HIV viral load and the immunological status (Noguera-Julian et al., 2016; Armstrong et al., 2018).

When evaluating the respective contribution of HIV and rectal infections on the microbial changes, we confirmed that CT or NG infections were the driver of the major shift toward anaerobes. Even if present, HIV infection affected the rectal microbiome composition differently than rectal infections.

We noticed that microbial composition of the rectal environment was affected by HIV only in *C. trachomatis* positive patients, whereas no differences were detected for NG, CT/NG and non-infected subjects.

However, it is necessary to observe that, in the CT group, significant differences in the microbial rectal habitat were detected on the basis of infecting serovars (L2 vs. non-L); thus, considering the strong association between HIV and LGV infection found in our setting, it is not possible to gain insight into their different contribution on the changes of the rectal microbiota composition. Further investigations will be needed to shed light on the dynamics that take place on the rectal mucosa during HIV and LGV infection.

Even in the case of well-controlled infections, HIV can perturb the equilibrium of the immune system, with various phenotypic and functional alterations on different cell populations (Mohan et al., 2014). Thus, we can speculate that HIV-positive and negative patients respond differently to the presence of a bacterial rectal infection. For that reason, HIV positivity affected the composition of the rectal microbiota only with an ongoing rectal infection, whereas in the control group no HIV-based effect was observed.

We are fully aware that several limitations could have affected our results. At first, sequencing of V3-V4 regions of the bacterial 16S rRNA gene allows a reliable taxonomic identification only down to the genus level; species-level identification would be helpful for a thorough comprehension of the dynamics of the rectal microenvironment, but this could be obtained only with the more in-depth and expensive technique of shotgun metagenome sequencing (i.e., direct sequencing of bacterial genomes in a community).

Moreover, more exhaustive information about the patients (e.g., use of PrEP; compliance and type of HIV antiretroviral therapy; number of sexual partners; date of the last sexual intercourse) would have been useful to find deeper correlations between clinical/behavioral factors and microbiome alterations.

Finally, this is a cross-sectional study with no sampling during the follow-up period; thus, further prospective investigations, including a larger number of subjects, will be essential to understand if the alterations of the rectal microbiota precede or follow chlamydial/gonococcal infections.

In conclusion, we elucidated some of the microbial changes that occur in the rectal mucosa during chlamydial/gonococcal infections in MSM. These data could open new perspectives for the control of sexually transmitted infections in this high-risk group (e.g., by using probiotics for prevention), as well as help in a thorough comprehension of the complex interactions between pathogens, commensals and the host.

## Data Availability Statement

The datasets generated for this study can be found in the NCBI Short Read Archive, http://www.ncbi.nlm.nih.gov/sra, Accession number: PRJNA545872.

## Ethics Statement

The studies involving human participants were reviewed and approved by Ethical Committee of St. Orsola-Malpighi Hospital (78/2017/U/Tess). The patients/participants provided their written informed consent to participate in this study.

## Author Contributions

AM and CF conceived and designed the study. AD'A and VG recruited volunteers and collected the samples. CF, CCo, and TC performed the experiments. CCe, LL, and MS analyzed the data. AM, CCo, and MR contributed reagents, materials, and analysis tools. AM, CF, CCe, CCo, and MS wrote the paper. All authors read, reviewed, and approved the final manuscript.

### Conflict of Interest

The authors declare that the research was conducted in the absence of any commercial or financial relationships that could be construed as a potential conflict of interest.

## References

[B1] Araújo-Pérez F. McCoy A. N. Okechukwu C. Carroll I. M. Smith K. M. Jeremiah K.. (2012). Differences in microbial signatures between rectal mucosal biopsies and rectal swabs. Gut Microbes 3, 530–535. 10.4161/gmic.22157 23060016 PMC3495790

[B2] Armstrong A. J. S. Shaffer M. Nusbacher N. M. Griesmer C. Fiorillo S. Schneider J. M.. (2018). An exploration of Prevotella-rich microbiomes in HIV and men who have sex with men. Microbiome 6:198. 10.1186/s40168-018-0580-7 30396369 PMC6219090

[B3] Bansal S. Nguyen J. P. Leligdowicz A. Zhang Y. Kain K. C. Ricciuto D. R. (2018). Rectal and naris swabs: practical and informative samples for analyzing the microbiota of critically Ill patients. mSphere 3:e00219–e00218. 10.1128/mSphere.00328-18 29898981 PMC6001609

[B4] Barbee L. A. Khosropour C. M. Dombrowksi J. C. Golden M. R. (2017). New human immunodeficiency virus diagnosis independently associated with rectal gonorrhea and chlamydia in men who have sex with men. Sex. Transm. Dis. 44, 385–389. 10.1097/OLQ.0000000000000614 28608786 PMC5481158

[B5] Bissessor M. Tabrizi S. N. Bradshaw C. S. Fairley C. K. Hocking J. S. Garland S. M.. (2016). The contribution of *Mycoplasma genitalium* to the aetiology of sexually acquired infectious proctitis in men who have sex with men. Clin. Microbiol. Infect. 22, 260–265. 10.1016/j.cmi.2015.11.016 26686807

[B6] Box G. E. P. Cox D. R. (2018). An analysis of transformations. J. R. Stat. Soc. Ser. B 26, 211–243. 10.1111/j.2517-6161.1964.tb00553.x

[B7] Budding A. E. Grasman M. E. Eck A. Bogaards J. A. Vandenbroucke-Grauls C. M. van Bodegraven A. A.. (2014). Rectal swabs for analysis of the intestinal microbiota. PLoS ONE 9:e101344. 10.1371/journal.pone.0101344 25020051 PMC4096398

[B8] Caporaso J. G. Kuczynski J. Stombaugh J. Bittinger K. Bushman F. D. Costello E. K.. (2010). QIIME allows analysis of high-throughput community sequencing data. Nat. Methods 7, 335–336. 10.1038/nmeth.f.303 20383131 PMC3156573

[B9] Chambers J. M. Freeny A. E. Heiberger R. M. (2017). Chapter 5: Analysis of variance; designed experiments, in Statistical Models, ed Hastie T. J. (New York, NY: Wadsworth & Brooks/Cole). 10.1201/9780203738535-5

[B10] Danby C. S. Cosentino L. A. Rabe L. K. Priest C. L. Damare K. C.. (2016). Patterns of extragenital chlamydia and gonorrhea in women and men who have sex with men reporting a history of receptive anal intercourse. Sex. Transm. Dis. 43, 105–109. 10.1097/OLQ.0000000000000384 26766527 PMC4955797

[B11] Foschi C. Gaspari V. Sgubbi P. Salvo M. D'Antuono A. Marangoni A. (2018a). Sexually transmitted rectal infections in a cohort of 'men having sex with men'. J. Med. Microbiol. 67, 1050–1057. 10.1099/jmm.0.000781 29927376

[B12] Foschi C. Laghi L. D'Antuono A. Gaspari V. Zhu C. Dellarosa N.. (2018b). Urine metabolome in women with *Chlamydia trachomatis* infection. PLoS ONE 13:e0194827. 10.1371/journal.pone.0194827 29566085 PMC5864028

[B13] Foschi C. Salvo M. D'Antuono A. Gaspari V. Banzola N. Cevenini R.. (2018c). Distribution of genital Mollicutes in the vaginal ecosystem of women with different clinical conditions. New Microbiol. 41, 225–229. 29620787

[B14] Goto Y. Kiyono H. (2012). Epithelial barrier: an interface for the cross-communication between gut flora and immune system. Immunol. Rev. 245, 147–163. 10.1111/j.1600-065X.2011.01078.x 22168418

[B15] Grov C. Cain D. Rendina H. J. Ventuneac A. Parsons J. T. (2016). Characteristics associated with urethral and rectal gonorrhea and chlamydia diagnoses in a US national sample of gay and bisexual men: results from the one thousand strong panel. Sex. Transm. Dis. 43, 165–171. 10.1097/OLQ.0000000000000410 26859803 PMC4748382

[B16] Heiligenberg M. Lutter R. Pajkrt D. Adams K. De Vries H. Heijman T.. (2013). Effect of HIV and chlamydia infection on rectal inflammation and cytokine concentrations in men who have sex with men. Clin. Vaccine Immunol. 20, 1517–1523. 10.1128/CVI.00763-12 23904458 PMC3807186

[B17] Igartua C. Davenport E. R. Gilad Y. Nicolae D. L. Pinto J. Ober C. (2017). Host genetic variation in mucosal immunity pathways influences the upper airway microbiome. Microbiome 5:16. 10.1186/s40168-016-0227-5 28143570 PMC5286564

[B18] Ingala M. R. Simmons N. B. Wultsch C. Krampis K. Speer K. A. Perkins S. L. (2018). Comparing microbiome sampling methods in a wild mammal: fecal and intestinal samples record different signals of host ecology, evolution. Front. Microbiol. 9:803. 10.3389/fmicb.2018.00803 29765359 PMC5938605

[B19] Kelley C. F. Kraft C. S. de Man T. J. Duphare C. Lee H. W. Yang J.. (2017). The rectal mucosa and condomless receptive anal intercourse in HIV-negative MSM: implications for HIV transmission and prevention. Mucosal Immunol. 10, 996–1007. 10.1038/mi.2016.97 27848950 PMC5433931

[B20] Klindworth A. Pruesse E. Schweer T. Peplies J. Quast C. Horn M.. (2013). Evaluation of general 16S ribosomal RNA gene PCR primers for classical and next-generation sequencing-based diversity studies. Nucleic Acids Res. 41:e1. 10.1093/nar/gks808 22933715 PMC3592464

[B21] Losurdo G. Iannone A. Contaldo A. Ierardi E. Di Leo A. Principi M. (2015). *Escherichia coli* Nissle 1917 in ulcerative colitis treatment: systematic review and meta-analysis. J. Gastrointestin. Liver Dis. 24, 499–505. 10.15403/jgld.2014.1121.244.ecn 26697577

[B22] Marangoni A. Foschi C. Nardini P. Compri M. Cevenini R. (2015). Evaluation of the Versant CT/GC DNA 1.0 assay (kPCR) for the detection of extra-genital *Chlamydia trachomatis* and *Neisseria gonorrhoeae* infections. PLoS ONE 10:e0120979. 10.1371/journal.pone.0120979 25799263 PMC4370730

[B23] Masella A. P. Bartram A. K. Truszkowski J. M. Brown D. G. Neufeld J. D. (2012). PANDAseq: paired-end assembler for illumina sequences. BMC Bioinformatics 13:31. 10.1186/1471-2105-13-31 22333067 PMC3471323

[B24] McHardy I. H. Li X. Tong M. Ruegger P. Jacobs J. Borneman J.. (2013). HIV Infection is associated with compositional and functional shifts in the rectal mucosal microbiota. Microbiome 1:26. 10.1186/2049-2618-1-26 24451087 PMC3971626

[B25] Mohan T. Bhatnagar S. Gupta D. L. Rao D. N. (2014). Current understanding of HIV-1 and T-cell adaptive immunity: progress to date. Microb. Pathog. 73, 60–69. 10.1016/j.micpath.2014.06.003 24930593

[B26] Mujugira A. Huang M. L. Selke S. Drolette L. Magaret A. S. Wald A. (2015). High rate of β-Globin DNA detection validates self-sampling in herpes simplex virus shedding studies. Sex. Transm. Dis. 42, 705–709. 10.1097/OLQ.0000000000000374 26562701 PMC4803496

[B27] Noguera-Julian M. Rocafort M. Guillén Y. Rivera J. Casadellà M. Nowak P.. (2016). Gut microbiota linked to sexual preference and HIV infection. EBioMedicine 5, 135–146. 10.1016/j.ebiom.2016.01.032 27077120 PMC4816837

[B28] Nowak P. Troseid M. Avershina E. Barqasho B. Neogi U. Holm K.. (2015). Gut microbiota diversity predicts immune status in HIV-1 infection. AIDS. 29, 2409–2418. 10.1097/QAD.0000000000000869 26355675

[B29] Nowak R. G. Bentzen S. M. Ravel J. Crowell T. A. Dauda W. Ma B.. (2017). Rectal microbiota among HIV-uninfected, untreated HIV, and treated HIV-infected in Nigeria. AIDS. 31, 857–862. 10.1097/QAD.0000000000001409 28118207 PMC5342931

[B30] Ohkusa T. Koido S. (2015). Intestinal microbiota and ulcerative colitis. J. Infect. Chemother. 21, 761–768. 10.1016/j.jiac.2015.07.010 26346678

[B31] Oksanen J. Blanchet F. G. Kindt R. Legendre P. Minchin P. R. O'Hara R. B. (2013). Package “Vegan.” R Package Version 2.0–10. Available online at: https://cran.r-project.org/src/contrib/Archive/vegan/vegan_2.0-10.tar.gz (accessed October 8, 2019).

[B32] Quillin S. J. Seifert H. S. (2018). *Neisseria gonorrhoeae* host adaptation and pathogenesis. Nat. Rev. Microbiol. 16, 226–240. 10.1038/nrmicro.2017.169 29430011 PMC6329377

[B33] Sassone-Corsi M. Raffatellu M. (2015). No vacancy: how beneficial microbes cooperate with immunity to provide colonization resistance to pathogens. J. Immunol. 194, 4081–4087. 10.4049/jimmunol.1403169 25888704 PMC4402713

[B34] Schäffler H. Breitrück A. (2018). *Clostridium difficile*—from colonization to infection. Front. Microbiol. 9:646. 10.3389/fmicb.2018.00646 29692762 PMC5902504

[B35] Sonnenborn U. (2016). *Escherichia coli* strain Nissle 1917-from bench to bedside and back: history of a special *Escherichia coli* strain with probiotic properties. FEMS Microbiol. Lett. 363:fnw212. 10.1093/femsle/fnw212 27619890

[B36] Sonnenburg J. L. Angenent L. T. Gordon J. I. (2004). Getting a grip on things: how do communities of bacterial symbionts become established in our intestine? Nat. Immunol. 5, 569–573. 10.1038/ni1079 15164016

[B37] Tang M. S. Poles J. Leung J. M. Wolff M. J. Davenport M. Lee S. C.. (2015). Inferred metagenomic comparison of mucosal and fecal microbiota from individuals undergoing routine screening colonoscopy reveals similar differences observed during active inflammation. Gut Microbes 6, 48–56. 10.1080/19490976.2014.1000080 25559083 PMC4615154

[B38] Tao G. Hoover K. W. Nye M. B. Peters P. Gift T. L. Peruvemba R.. (2016). Rectal infection with *Neisseria gonorrhoeae* and *Chlamydia trachomatis* in men in the United States. Clin. Infect. Dis. 63, 1325–1331. 10.1093/cid/ciw594 27572098

[B39] Unemo M. Shafer W. M. (2014). Antimicrobial resistance in *Neisseria gonorrhoeae* in the 21st century: past, evolution, and future. Clin. Microbiol. Rev. 27, 587–613. 10.1128/CMR.00010-14 24982323 PMC4135894

[B40] Vasilevsky S. Greub G. Nardelli-Haefliger D. Baud D. (2014). Genital Chlamydia trachomatis: understanding the roles of innate and adaptive immunity in vaccine research. Clin. Microbiol. Rev. 27, 346–370. 10.1128/CMR.00105-13 24696438 PMC3993100

[B41] Wang Q. Garrity G. M. Tiedje J. M. Cole J. R. (2007). Naive Bayesian classifier for rapid assignment of rRNA sequences into the new bacterial taxonomy. Appl. Environ. Microbiol. 73, 5261–5267. 10.1128/AEM.00062-07 17586664 PMC1950982

[B42] Zhang N. Li T. Z. Zheng K. Mou D. L. Liang L. C. Zhang T.. (2018). Use of rectal swab samples for analysis of the intestinal microbiome in children. Chin. Med. J. 131, 492–494. 10.4103/0366-6999.225065 29451159 PMC5830839

