# Supplementary material for: Rectal Microbiota Associated With Chlamydia trachomatis and Neisseria gonorrhoeae Infections in Men Having Sex With Other Men
Source: Front Cell Infect Microbiol. 2019 Oct 18;9:358. doi: 10.3389/fcimb.2019.00358 (PMC6813206; doi:10.3389/fcimb.2019.00358)
Supplement: Supplementary file 1 [file Table_1.DOCX]

**Supplementary materials**

**Table S1. Alpha diversity comparisons between each group of patients for the metric used.** For each patient group, average ± standard deviation are reported. p-values of the permutation-based test for assessing differences in alpha-diversity between each infection group and non-infected subjects are reported within brackets.

|  | **No infection** | **CT** | **NG** | **CT/NG** |
| --- | --- | --- | --- | --- |
| **Chao1** | 2836.22 ± 1377.64 | 3490.56 ± 1363.43  (0.31) | 2951.26 ±1266.49  (1.0) | 3076.88 ± 1668.06  (1.0) |
| **Observed Species** | 1106.88 ± 488.8 | 1346.18 ± 448.42  (0.16) | 1224.18 ± 450.81  (1.0) | 1276.79 ± 633.42  (1.0) |
| **Shannon** | 5.44 ± 1.2 | 6.14 ± 0.7  (0.054) | 5.68 ± 0.87  (1.0) | 5.7 ± 1.37  (1.0) |
| **Faith’s phylogenetic diversity** | 68.37 ± 23.93 | 80.96 ± 22.28  (0.084) | 74.41 ± 21.9  (1.0) | 78.27 ± 31.39  (1.0) |
| **Good’s coverage** | 0.96 ± 0.02 | 0.95 ± 0.02  (0.198) | 0.95 ± 0.02  (1.0) | 0.95 ± 0.03  (1.0) |

**Table S2. Average relative abundance of main microbial phyla.** Data are expressed as mean ± standard deviation (stdev). For each phylum, significant p-values of the non-parametric Kruskal-Wallis test and of pairwise comparison (Dunn’s test) versus non-infected patients are reported. Only phylum present at relative abundances >1% on average in at least one subgroup are listed.

|  | **Average (stdev)** | | | |  | **Kruskal-Wallis Dunn’s test** | | | |
| --- | --- | --- | --- | --- | --- | --- | --- | --- | --- |
|  |  |  |  |  |  |  |  |  |  |
|  | **No**  **Infection** | **CT** | **NG** | **CT/NG** |  | **K-W p-value** | **CT** | **NG** | **CT/NG** |
|  |  |  |  |  |  |  |  |  |  |
| ***Firmicutes*** | 36.36 (14.43) | 39.65 (10.23) | 36.81 (9.13) | 36.86 (10.33) |  | -- | -- | -- | -- |
| ***Bacteroidetes*** | 28.05 (13.09) | 30.44 (10.39) | 33.29 (10.08) | 28.11 (13.50) |  | -- | -- | -- | -- |
| ***Proteobacteria*** | 24.92 (21.37) | 16.00 (14.26) | 19.65 (10.60) | 24.32 (17.37) |  | -- | -- | -- | -- |
| ***Fusobacteria*** | 5.68 (8.97) | 8.10 (8.24) | 6.74 (6.83) | 7.10 (9.03) |  | -- | -- | -- | -- |
| ***Actinobacteria*** | 3.54 (7.43) | 3.27 (5.10) | 1.44 (1.34) | 1.59 (1.25) |  | -- | -- | -- | -- |
| ***Chlamydiae*** | 0.01 (0.03) | 1.05 (1.85) | 0.00 (0.01) | 0.90 (1.87) |  | <0.001 | <0.001 | -- | <0.001 |
| ***Tenericutes*** | 0.20 (0.56) | 0.41 (0.98) | 1.12 (4.37) | 0.57 (1.29) |  | 0.038 | 0.040 | -- | 0.018 |
| **Other** | 1.24 | 1.08 | 0.95 | 0.55 |  |  |  |  |  |

**Table S3. Average relative abundance of main microbial families.** Data are expressed as mean ± standard deviation (stdev). For each family, significant p-values of the non-parametric Kruskal-Wallis test and of pairwise comparison (Dunn’s test) versus non-infected patients are reported. Only families present at relative abundances >1% on average in at least one subgroup are listed.

|  |  |  |  |  |  |  |  |  |  |
| --- | --- | --- | --- | --- | --- | --- | --- | --- | --- |
|  | **Average (stdev)** | | | |  | **Kruskal-Wallis Dunn’s test** | | | |
|  |  |  |  |  |  |  |  |  |  |
|  | **No**  **Infection** | **CT** | **NG** | **CT/NG** |  | **K-W p-value** | **CT** | **NG** | **CT/NG** |
|  |  |  |  |  |  |  |  |  |  |
| ***Prevotellaceae*** | 20.59 (11.62) | 22.99 (10.18) | 24.41 (7.97) | 19.82 (9.84) |  | -- | -- | -- | -- |
| ***Enterobacteriaceae*** | 16.31 (22.98) | 7.85  (14.78) | 6.96 (10.02) | 5.88  (6.98) |  | 0.043 | 0.007 | 0.017 | -- |
| ***Ruminococcaceae*** | 9.67  (9.31) | 8.52  (6.57) | 7.26  (7.10) | 8.30  (8.57) |  | -- | -- | -- | -- |
| ***[Tissierellaceae]*** | 4.68  (4.78) | 8.03  (6.79) | 6.88  (6.11) | 7.60  (5.78) |  | 0.027 | 0.004 | -- | 0.014 |
| ***Veillonellaceae*** | 5.82  (4.95) | 6.04  (3.18) | 5.83  (3.29) | 5.14  (3.72) |  | -- | -- | -- | -- |
| ***Lachnospiraceae*** | 5.44  (4.36) | 6.24  (4.89) | 4.62  (4.89) | 3.67  (3.17) |  | -- | -- | -- | -- |
| ***Fusobacteriaceae*** | 4.02  (6.07) | 5.82  (6.03) | 4.89  (6.01) | 4.48  (5.02) |  | -- | -- | -- | -- |
| ***Bacteroidaceae*** | 3.33  (4.32) | 2.54  (3.82) | 4.50  (4.92) | 3.45  (5.67) |  | -- | -- | -- | -- |
| ***Pasteurellaceae*** | 3.44  (7.04) | 0.98  (2.20) | 2.24  (6.39) | 4.33 (10.50) |  | -- | -- | -- | -- |
| ***Erysipelotrichaceae*** | 2.39  (2.84) | 3.29  (2.95) | 1.27  (1.01) | 2.09  (2.36) |  | -- | -- | -- | -- |
| ***Succinivibrionaceae*** | 1.93  (4.44) | 3.06  (4.64) | 1.56  (2.95) | 3.19  (4.71) |  | -- | -- | -- | -- |
| ***Leptotrichiaceae*** | 1.64  (4.77) | 2.26  (4.40) | 1.83  (2.88) | 2.60  (5.87) |  | -- | -- | -- | -- |
| ***Streptococcaceae*** | 2.04  (4.89) | 1.25  (2.36) | 2.02  (3.77) | 3.31  (6.37) |  | -- | -- | -- | -- |
| ***Campylobacteraceae*** | 1.67  (2.81) | 2.06  (2.60) | 2.42  (2.49) | 2.07  (2.73) |  | -- | -- | -- | -- |
| ***Porphyromonadaceae*** | 1.39  (1.76) | 2.38  (3.72) | 2.21  (2.75) | 2.37  (2.82) |  | -- | -- | -- | -- |
| ***Peptostreptococcaceae*** | 0.71  (1.18) | 2.06  (2.89) | 1.99  (1.79) | 1.71  (1.18) |  | <0.001 | <0.001 | <0.001 | <0.001 |
| ***[Paraprevotellaceae]*** | 1.10  (1.55) | 1.32  (1.29) | 0.68  (1.29) | 1.34  (1.96) |  | -- | -- | -- | -- |
| ***Carnobacteriaceae*** | 0.48  (0.94) | 1.11  (2.14) | 1.24  (2.51) | 1.01  (1.74) |  | 0.025 | 0.010 | -- | 0.006 |
| ***Neisseriaceae*** | 0.14  (0.50) | 0.07  (0.12) | 4.99  (7.18) | 7.28  (14.9) |  | <0.001 | -- | <0.001 | <0.001 |
| ***Staphylococcaceae*** | 2.35  (6.36) | 0.72  (2.48) | 1.38  (4.86) | 0.66  (1.70) |  | -- | -- | -- | -- |
| ***Corynebacteriaceae*** | 1.73  (4.41) | 0.97  (2.01) | 0.26  (0.39) | 0.35  (0.57) |  | -- | -- | -- | -- |
| ***Enterococcaceae*** | 0.74  (2.41) | 0.09  (0.15) | 1.22  (3.10) | 0.36  (0.76) |  | -- | -- | -- | -- |
| ***Chlamydiaceae*** | 0.01  (0.03) | 1.05  (1.85) | 0.00  (0.00) | 0.90  (1.87) |  | <0.001 | <0.001 | 0.014 | <0.001 |
| ***Mycoplasmataceae*** | 0.16  (0.54) | 0.38  (0.98) | 1.12  (4.37) | 0.57  (1.30) |  | 0.031 | 0.018 | -- | 0.012 |
| ***Lactobacillaceae*** | 0.02  (0.06) | 0.10  (0.44) | 0.00  (0.00) | 1.14  (4.79) |  | -- | -- | -- | -- |
| ***Leuconostocaceae*** | 0.00  (0.00) | 0.00  (0.00) | 1.22  (5.00) | 0.10  (0.40) |  | 0.008 | -- | -- | <0.001 |
| **Other** | 8.18 | 8.83 | 6.97 | 6.27 |  |  |  |  |  |

**Table S4. Multivariate model (two-way ANOVA) to investigate the respective contributions of HIV and CT/NG infections on the rectal microbiome changes.** For each phylum, family and genus, p-values are reported. Statistically significant results (p<0.05) are indicated by an asterisk.

| **Phyla** | **Rectal infections** | **HIV infection** |
| --- | --- | --- |
| Firmicutes | 0.17 | 0.33 |
| Bacteroidetes | 0.24 | 0.54 |
| Proteobacteria | 0.10 | 0.93 |
| Fusobacteria | 0.49 | 0.19 |
| Actinobacteria | 0.33 | 0.47 |
| Tenericutes | 0.05 | 0.82 |

| **Families** | **Rectal infections** | **HIV infection** |
| --- | --- | --- |
| *Prevotellaceae* | 0.21 | 0.59 |
| *Enterobacteriaceae* | 0.02* | 0.66 |
| *Ruminococcaceae* | 0.44 | 0.14 |
| *Tissierellaceae* | 0.005* | 0.32 |
| *Veillonellaceae* | 0.23 | 0.17 |
| *Lachnospiraceae* | 0.10 | 0.37 |
| *Fusobacteriaceae* | 0.46 | 0.29 |
| *Bacteroidaceae* | 0.83 | 0.16 |
| *Pasteurellaceae* | 0.39 | 0.54 |
| *Erysipelotrichaceae* | 0.15 | 0.85 |
| *Succinivibrionaceae* | 0.27 | 0.97 |
| *Leptotrichiaceae* | 0.64 | 0.06 |
| *Streptococcaceae* | 0.96 | 0.58 |
| *Campylobacteraceae* | 0.30 | 0.70 |
| *Porphyromonadaceae* | 0.80 | 0.67 |
| *Peptostreptococcaceae* | 0.00001* | 0.87 |
| *Staphylococcaceae* | 0.03* | 0.90 |
| *Paraprevotellaceae* | 0.15 | 0.95 |
| *Corynebacteriaceae* | 0.20 | 0.06 |
| *Carnobacteriaceae* | 0.08 | 0.02* |
| *Enterococcaceae* | 0.22 | 0.04* |
| *Mycoplasmataceae* | 0.04* | 0.5 |
| *Lactobacillaceae* | 0.17 | 0.16 |
| *Leuconostocaceae* | 0.004* | 0.01* |

| **Genera** | **Rectal infections** | **HIV infection** |
| --- | --- | --- |
| *Prevotella* | 0.23 | 0.57 |
| *Escherichia* | 0.002* | 0.38 |
| *Fusobacterium* | 0.49 | 0.37 |
| *Faecalibacterium* | 0.57 | 0.29 |
| *Bacteroides* | 0.83 | 0.16 |
| *Dialister* | 0.06 | 0.02* |
| *Oscillospira* | 0.40 | 0.04* |
| *Succinivibrio* | 0.30 | 0.97 |
| *Streptococcus* | 0.95 | 0.57 |
| *Campylobacter* | 0.30 | 0.70 |
| *Sneathia* | 0.68 | 0.08 |
| *Haemophilus* | 0.62 | 0.23 |
| *Finegoldia* | 0.85 | 0.51 |
| *Peptoniphilus* | 0.0003* | 0.19 |
| *Porphyromonas* | 0.27 | 0.14 |
| *Uncl. Ruminococcaceae* | 0.16 | 0.33 |
| *Peptostreptococcus* | 0.00009* | 0.67 |
| *Staphylococcus* | 0.03* | 0.90 |
| *Uncl. Lachnospiraceae* | 0.09 | 0.06 |
| *Corynebacterium* | 0.20 | 0.06 |
| *Enterobacter* | 0.19 | 0.76 |
| *Roseburia* | 0.07 | 0.11 |
| *Catenibacterium* | 0.80 | 0.59 |
| *Granulicatella* | 0.05 | 0.03* |
| *Uncl. Fusobacteriaceae* | 0.10 | 0.26 |
| *Parvimonas* | 0.005* | 0.21 |
| *Pasteurella* | 0.008* | 0.89 |
| *Enterococcus* | 0.23 | 0.11 |
| *Actinobacillus* | 0.19 | 0.08 |
| *Mycoplasma* | 0.20 | 0.53 |
| *Lactobacillus* | 0.21 | 0.13 |
| *Weissella* | 0.30 | 0.03* |

**Figure S1. Weighted beta-diversity of the rectal microbiota.** Principal Coordinate Analysis (PCoA) plot based on weighted Unifrac distance (beta-diversity). Each point corresponds to a sample. For each experimental group, the SEM-based confidence ellipse and the average value centroid are depicted. The second and third principal coordinates are represented.

**
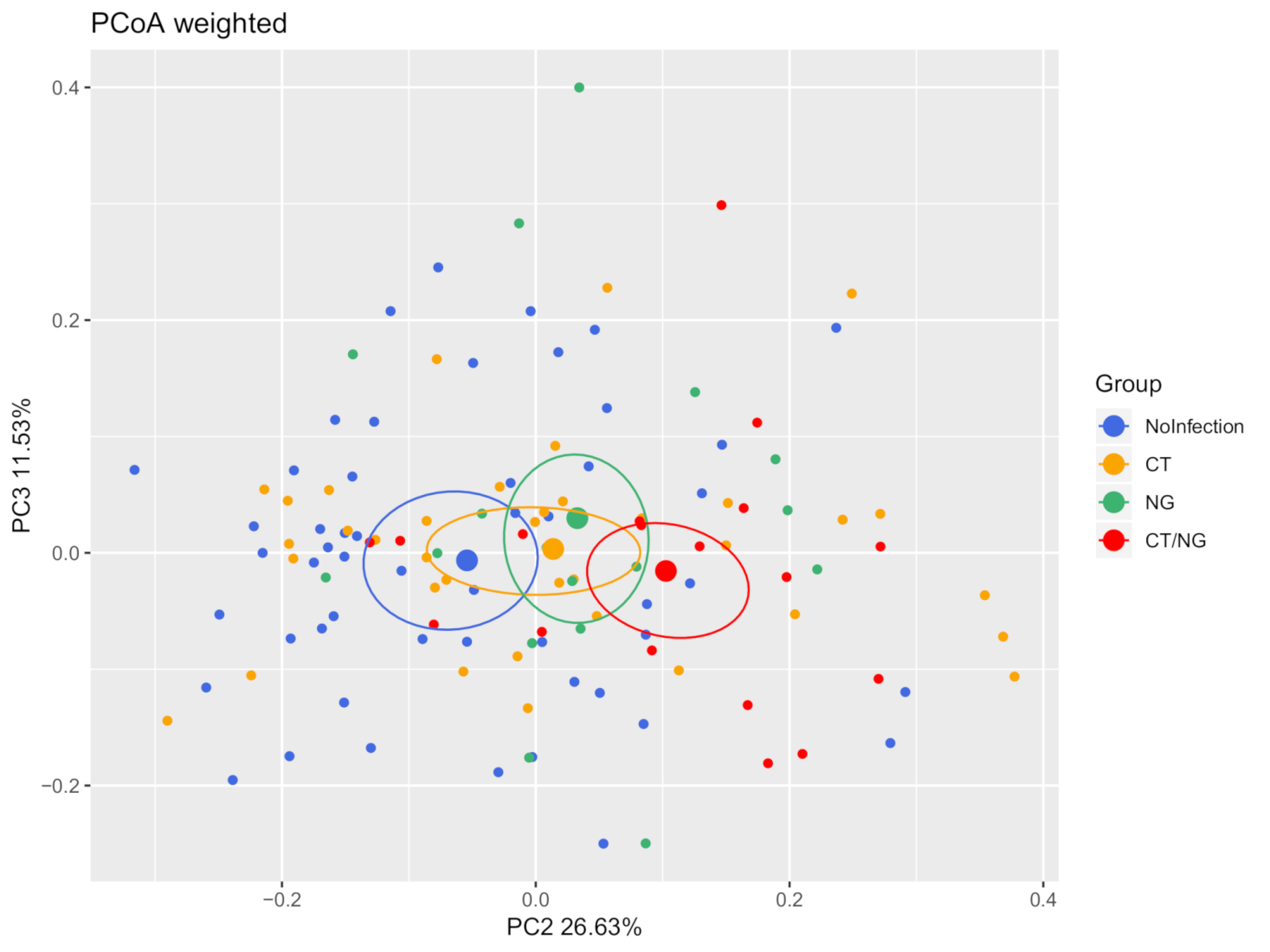
**

**Figure S2. Taxonomic composition of the rectal microbiota for each single patient.** Stacked bar charts of taxonomy relative abundances at the genus level for the different subgroups, stratified by the HIV-status. A: subjects with no rectal infection; B: patients with *C. trachomatis* infection (CT), divided in L2 serovar and non-L serovars; C: patients with *N. gonorrhoeae* (NG) infection; D: patients with contemporary positivity for both pathogens (CT/NG). Patients with unknown HIV status were not included.

**
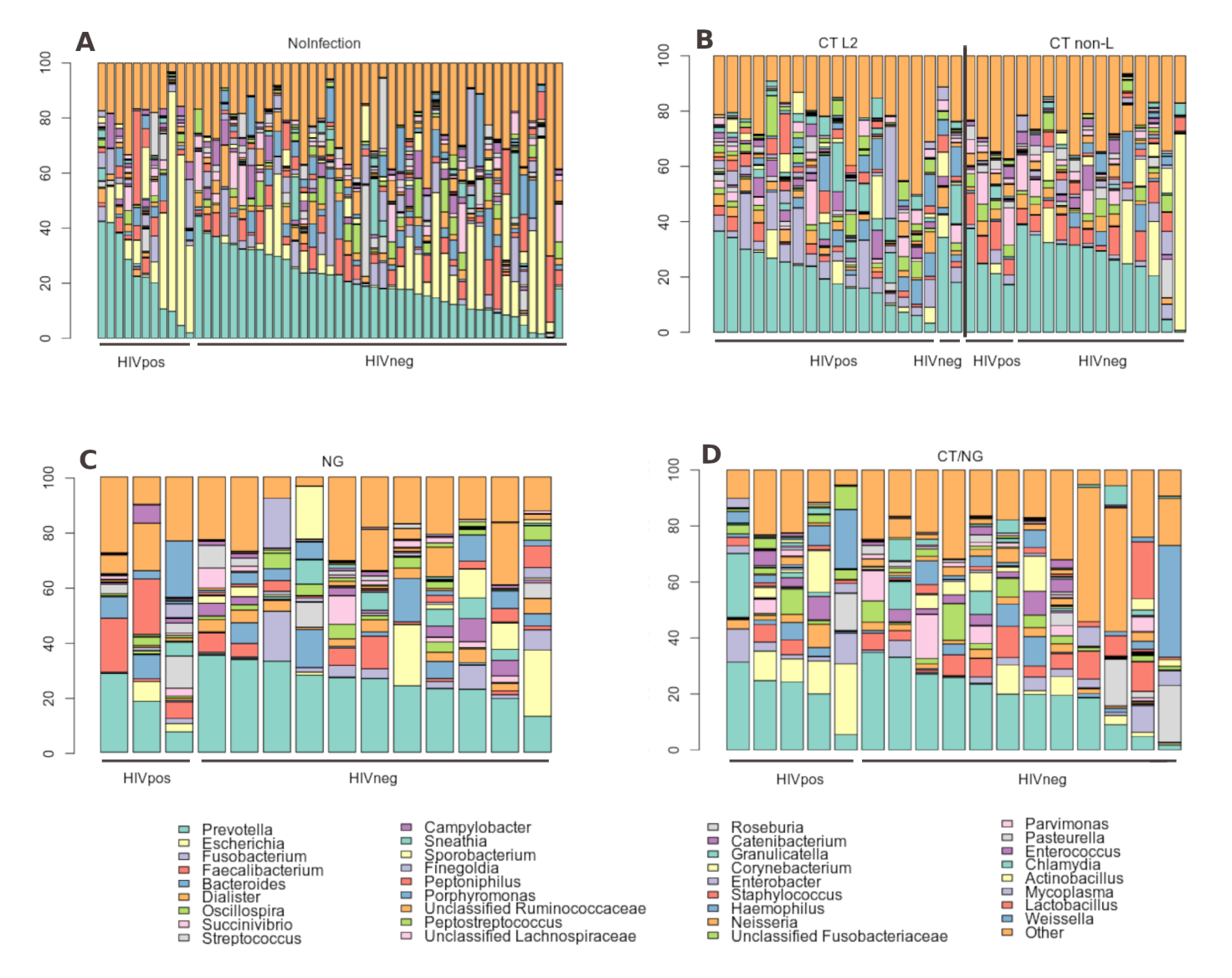
**
